# Supplementary material for: Analysis of PPARγ Signaling Activity in Psoriasis
Source: Int J Mol Sci. 2021 Aug 10;22(16):8603. doi: 10.3390/ijms22168603 (PMC8395241; doi:10.3390/ijms22168603)
Supplement: Supplementary file 1 [file ijms-22-08603-s001.zip › Supplemental materials_Analysis of PPARg signaling activity in psoriasis/Pathway models/Models images and html files/Interleukin-17 and interleukin-22 signaling in psoriasis/10368.html]

CXCL5


# Protein CXCL5

|  |  |
| --- | --- |
| URN | urn:agi-llid:6374 |
| Total Entities | 0 |
| Connectivity | 1577 |
| Name | CXCL5 |
| Description | chemokine (C-X-C motif) ligand 5 |
| Notes | This gene encodes a protein that is a member of the CXC subfamily of chemokines. Chemokines, which recruit and activate leukocytes, are classified by function (inflammatory or homeostatic) or by structure. This protein is proposed to bind the G-protein coupled receptor chemokine (C-X-C motif) receptor 2 to recruit neutrophils, to promote angiogenesis and to remodel connective tissues. This protein is thought to play a role in cancer cell proliferation, migration, and invasion. [provided by RefSeq, May 2013] |
| Primary Cell Localization | Extracellular |
| Class | Ligand |

---

|  |  |
| --- | --- |
| Pathway | CXC Chemokine Receptor Signaling |
|  | Neutrophil Chemotaxis |
|  | Proteins Involved in Atherosclerosis |
|  | Proteins Involved in Myocardial Ischemia |
|  | IL17 Signaling in Psoriasis |
|  | Adipokines and Cytokines in Insulin Resistance in Skeletal Muscles |
|  | Insulin Resistance in Myocytes Induced by Obesity |
|  | Adipose Tissue Inflammation Provokes Insulin Resistance |
|  | Cytokines Trigger Otitis Media |
|  | Endothelial Cell Dysfunction in Pyelonephritis |
|  | CXCL5 Expression Targets |
|  | Immunoglobulin Superfamily -> Expression Targets in Lymphoid System and Blood |
|  | pprarg neg, uknown targets, ps-positive |
|  | prarg neg,ukn expres targets, ps-positive |
|  | PPARG negative targets |
|  | pparg expr target\_regulators |
|  | Neighbors of disease exacerbation |
|  | Neighbors of tumor progression |
|  | Neighbors of cancer progression |
|  | Neighbors of overall survival |
|  | Neighbors of tumor size |
|  | Neighbors of mortality |
|  | Neighbors of survival time |
|  | Neighbors of insulin sensitivity |
|  | Neighbors of bacterial load |
|  | Neighbors of therapeutic efficacy |
|  | Neighbors of clinical stage |
|  | Neighbors of microvessel density |
|  | Neighbors of neutrophil count |
|  | Neighbors of exhaustion |
|  | Neighbors of tumor grade |
|  | Neighbors of body mass index |
|  | Neighbors of carotid intima-media thickness |
|  | Neighbors of disease exacerbation |
|  | Neighbors of tumor progression |
|  | Neighbors of cancer progression |
|  | Neighbors of overall survival |
|  | Neighbors of tumor size |
|  | Neighbors of mortality |
|  | Neighbors of survival time |
|  | Neighbors of insulin sensitivity |
|  | Neighbors of bacterial load |
|  | Neighbors of therapeutic efficacy |
|  | Neighbors of clinical stage |
|  | Neighbors of microvessel density |
|  | Neighbors of neutrophil count |
|  | Neighbors of exhaustion |
|  | Neighbors of tumor grade |
|  | Neighbors of body mass index |
|  | Neighbors of carotid intima-media thickness |
|  | Neighbors of immune response |
|  | Neighbors of inflammatory response |
|  | Neighbors of wound healing |
|  | Neighbors of angiogenesis |
|  | Neighbors of innate immune response |
|  | Neighbors of cell function |
|  | Neighbors of macrophage activation |
|  | Neighbors of cell formation |
|  | Neighbors of osteoclast development |
|  | Neighbors of epithelial to mesenchymal transition |
|  | Neighbors of cell proliferative response |
|  | Neighbors of chemotaxis |
|  | Neighbors of cell survival |
|  | Neighbors of cell phenotype |
|  | Neighbors of tissue remodeling |
|  | Neighbors of endothelial cell proliferation |
|  | Neighbors of tumor growth |
|  | Neighbors of dendritic cell development |
|  | Neighbors of tissue repair |
|  | Neighbors of cell count |
|  | Neighbors of cell infiltration |
|  | Neighbors of lumen formation |
|  | Neighbors of endothelial cell migration |
|  | Neighbors of lipid storage |
|  | Neighbors of cell motility |
|  | Neighbors of microglial activation |
|  | Neighbors of neutrophil recruitment |
|  | Neighbors of cell migration |
|  | Neighbors of cancer growth |
|  | Neighbors of hepatic regeneration |
|  | Neighbors of transendothelial migration |
|  | Neighbors of leukocyte migration |
|  | Neighbors of leukocyte recruitment |
|  | Neighbors of monocyte recruitment |
|  | Neighbors of cell invasion |
|  | Neighbors of hemopoiesis |
|  | Neighbors of T-cell function |
|  | Neighbors of lung development |
|  | Neighbors of tumor immunity |
|  | Neighbors of hepatocyte proliferation |
|  | Neighbors of cell growth |
|  | Neighbors of ROS generation |
|  | Neighbors of cancer cell growth |
|  | Neighbors of sensitization |
|  | Neighbors of hemato-encephalic barrier |
|  | Neighbors of cellular senescence |
|  | Neighbors of colony formation |
|  | Neighbors of cell differentiation |
|  | Neighbors of inflammatory disease |
|  | Neighbors of rheumatoid arthritis |
|  | Neighbors of atherosclerosis |
|  | Neighbors of fibrosis |
|  | Neighbors of colitis |
|  | Neighbors of arthritis |
|  | Neighbors of inflammatory bowel disease |
|  | Neighbors of injury |
|  | Neighbors of atherogenesis |
|  | Neighbors of metastasis |
|  | Neighbors of carcinogenesis |
|  | Neighbors of chronic inflammation |
|  | Neighbors of inflammation |
|  | Neighbors of tumor microenvironment |
|  | Neighbors of pathological angiogenesis |
|  | Neighbors of obesity |
|  | Neighbors of diabetes mellitus |
|  | Neighbors of asthma |
|  | Neighbors of pneumonia |
|  | Neighbors of hyperplasia |
|  | Neighbors of myocardial infarction |
|  | Neighbors of reperfusion injury |
|  | Neighbors of airway inflammation |
|  | Neighbors of insulin resistance |
|  | Neighbors of infection |
|  | Neighbors of renal injury |
|  | Neighbors of liver injury |
|  | Neighbors of neuroinflammation |
|  | Neighbors of death |
|  | Neighbors of dermatitis |
|  | Neighbors of neutrophil infiltration |
|  | Neighbors of diabetic nephropathy |
|  | Neighbors of lung injury |
|  | Neighbors of immunopathology |
|  | Neighbors of hepatocellular carcinoma |
|  | Neighbors of acute lung injury |
|  | Neighbors of gastric cancer |
|  | Neighbors of colorectal cancer |
|  | Neighbors of breast cancer |
|  | Neighbors of ischemia |
|  | Neighbors of melanoma |
|  | Neighbors of cartilage degeneration |
|  | Neighbors of cardiovascular disease |
|  | Neighbors of cancer |
|  | Neighbors of type 2 diabetes |
|  | Neighbors of neoplasm |
|  | Neighbors of endometriosis |
|  | Neighbors of synovitis |
|  | Neighbors of coronary artery disease |
|  | Neighbors of rupture |
|  | Neighbors of prostate cancer |
|  | Neighbors of allograft rejection |
|  | Neighbors of epithelial cell |
|  | Neighbors of keratinocyte |
|  | Neighbors of umbilical vein endothelial cell |
|  | Neighbors of monocyte |
|  | Neighbors of endometrial stromal cell |
|  | Neighbors of alveolar macrophage |
|  | Neighbors of synoviocyte |
|  | Neighbors of macrophage |
|  | Neighbors of activated monocyte |
|  | Neighbors of fibroblast |
|  | Neighbors of airway epithelial cell |
|  | Neighbors of PBMC |
|  | Neighbors of stromal cell |
|  | Neighbors of osteoblast |
|  | Neighbors of pneumocyte |
|  | Neighbors of microglia |
|  | Neighbors of intestine epithelium cell |
|  | Neighbors of mononuclear cell |
|  | Neighbors of endothelial cell |
|  | Neighbors of dendritic cell |
|  | Neighbors of inflammatory cell |
|  | Neighbors of bone marrow macrophage |
|  | Neighbors of peritoneal macrophage |
|  | Neighbors of neutrophil |
|  | Neighbors of astrocyte |
|  | Neighbors of mesothelial cell |
|  | Neighbors of capillary endothelial cell |
|  | Neighbors of immunocompetent cell |
|  | Neighbors of cardiac myocyte |
|  | Neighbors of cancer-associated fibroblast |
|  | Neighbors of myeloid cell |
|  | Neighbors of mesenchymal stroma cell |
|  | Neighbors of stem cell |
|  | Neighbors of tumor infiltrating macrophage |
|  | Neighbors of leukocyte |
|  | Neighbors of M1 macrophage |
|  | Neighbors of myofibroblast |
|  | Neighbors of mast cell |
|  | Neighbors of neuroglia |
|  | Neighbors of podocyte |
|  | Neighbors of phagocyte |
|  | Neighbors of retina cell |
|  | Neighbors of lung |
|  | Neighbors of liver |
|  | Neighbors of lymph node |
|  | Neighbors of colon |
|  | Neighbors of bone |
|  | Neighbors of synovial membrane |
|  | Neighbors of uterus |
|  | Neighbors of brain |
|  | Neighbors of islets of Langerhans |
|  | Neighbors of spleen |
|  | Neighbors of peritoneum |
|  | Neighbors of nasopharynx |
|  | Neighbors of NF-kB family |
|  | Neighbors of JNK |
|  | Neighbors of IL1 family |
|  | Neighbors of NFKBI |
|  | Neighbors of PI3K |
|  | Neighbors of mitogen-activated protein kinase |
|  | Neighbors of IFNAR ligand |
|  | Neighbors of TLR |
|  | Neighbors of cytokine |
|  | Neighbors of interleukin |
|  | Neighbors of PDGF |
|  | Neighbors of ERK1/2 |
|  | Neighbors of PKA |
|  | Neighbors of NF-AT family |
|  | Neighbors of IKBK |
|  | Neighbors of p38 |
|  | Neighbors of inflammatory cytokine |
|  | Neighbors of IL23 |
|  | Neighbors of HIF-1 |
|  | Neighbors of IL12 |
|  | Neighbors of fibrinogen |
|  | Neighbors of mitogen-activated protein kinase kinase |
|  | Neighbors of proteasome endopeptidase complex |
|  | Neighbors of SAPK |
|  | Neighbors of AHR subfamily |
|  | Neighbors of ITG |
|  | Neighbors of TNF-alpha receptor |
|  | comon ps\_pos, pprarg\_neg targets |
|  | 2\_Interleukin-17 and interleukin-22 signaling in psoriasis |
|  | 2\_The role of endothelial cells in pyelonephritis |
|  | 4\_1\_Polymorphisms associated with inflammatory bowel diseases |
|  | 4\_2\_Insulin resistance: inflammation-related insulin resistance |
|  | drug-target moltransport |
|  | Interleukin-17 and interleukin-22 signaling in psoriasis |

---

|  |  |
| --- | --- |
| Group | Chemokines, Growth Factors and Receptors Involved in Cancer Cells Chemotaxis |

---

|  |  |
| --- | --- |
| MedScan ID | 6374 |

---

|  |  |
| --- | --- |
| LocusLink ID | 6374 |

---

|  |  |
| --- | --- |
| Alias | small inducible cytokine B5 |
|  | neutrophil-activating protein 78 |
|  | C-X-C motif chemokine ligand 5 |
|  | small inducible cytokine subfamily B (Cys-X-Cys), member 5 (epithelial-derived neutrophil-activating peptide 78) |
|  | chemokine (C-X-C motif) ligand V |
|  | small inducible cytokine subfamily B (Cys-X-Cys), member 5 |
|  | ENA-78 |
|  | small inducible cytokine subfamily B (Cys-X-Cys), member V |
|  | ENA-78(1-78) |
|  | SCYB5 |
|  | CXC ligand 5 |
|  | epithelial derived neutrophil activating factor 78 |
|  | neutrophil activated peptide 78 |
|  | C-X-C motif chemokine 5 |
|  | SZ05\_HUMAN |
|  | neutophil-activating peptide 78 |
|  | CXC chemokine LIX |
|  | Epithelial-derived neutrophil-activating protein 78 |
|  | CXCL5 chemokine |
|  | neutrophil activating peptide 78 |
|  | AMCF-IIs |
|  | epithelial-derived neutrophil-activating peptide 78 |
|  | CXCL5 |
|  | LOC60665 |
|  | epithelial neutrophil activating factor 78 |
|  | chemokine (C-X-C motif) ligand 5 |
|  | SZ05 |
|  | neutrophil-activating peptide ENA-78 |
|  | LIX |
|  | chemokine CXCL5 |
|  | chemokine CXC ligand 5 |
|  | CXC chemokine ligand 5 |
|  | AMCF-II |

---

|  |  |
| --- | --- |
| GO ID | 0008009 |
|  | 0007186 |
|  | 0061844 |
|  | 0007267 |
|  | 0071222 |
|  | 0070098 |
|  | 0006935 |
|  | 0006955 |
|  | 0006954 |
|  | 0030595 |
|  | 0030593 |
|  | 0008284 |
|  | 0007165 |
|  | 0005576 |
|  | 0005615 |
|  | 0045236 |
|  | 0060326 |
|  | 0002446 |
|  | 0002690 |
|  | 0032496 |

---

|  |  |
| --- | --- |
| KEGG ID | hsa:6374 |

---

|  |  |
| --- | --- |
| Organism | Homo sapiens {Organism urn:agi-taxid:9606} |
|  | Homo sapiens |

---

|  |  |
| --- | --- |
| OMIM ID | 600324 |

---

|  |  |
| --- | --- |
| Hugo ID | 10642 |
|  | HGNC:10642 |

---

|  |  |
| --- | --- |
| Human chromosome position | 4q13.3 |

---

|  |  |
| --- | --- |
| Swiss-Prot Accession | P42830 |
|  | Q6I9S7 |
|  | P42830.1 |
|  | Q96QE1 |

---

|  |  |
| --- | --- |
| PIR ID | JC2433 |

---

|  |  |
| --- | --- |
| GenBank ID | NC\_000004 |
|  | NM\_002994 |
|  | NP\_002985 |
|  | AC097709 |
|  | AAY41005 |
|  | AF349466 |
|  | AAK29641 |
|  | AJ315731 |
|  | AJ315732 |
|  | CAC42884 |
|  | CH471057 |
|  | EAX05696 |
|  | FB342965 |
|  | CAR81483 |
|  | FB711088 |
|  | CAS91499 |
|  | L37036 |
|  | AAA86426 |
|  | U12709 |
|  | AAA62475 |
|  | AK026546 |
|  | AK312128 |
|  | BAG35064 |
|  | BC008376 |
|  | AAH08376 |
|  | BT006807 |
|  | AAP35453 |
|  | BU169770 |
|  | BU626570 |
|  | CB529782 |
|  | CR457428 |
|  | CAG33709 |
|  | DN992282 |
|  | X78686 |
|  | CAA55355 |
|  | P42830 |
|  | NC\_018915 |
|  | AMYH02009472 |
|  | DQ895547 |
|  | ABM86473 |
|  | EU176356 |
|  | ABW03807 |
|  | AC\_000136 |
|  | ABBA01022994 |

---

|  |  |
| --- | --- |
| Swiss-Prot ID | CXCL5\_HUMAN |

---

|  |  |
| --- | --- |
| Cell Localization | Secreted |

---

|  |  |
| --- | --- |
| Ensembl ID | ENSG00000163735 |
|  | ENSP00000296027.4 |
|  | ENST00000296027.5 |
|  | ENSP00000296027 |
|  | ENST00000296027 |

---

|  |  |
| --- | --- |
| Unigene ID | Hs.89714 |

---

|  |  |
| --- | --- |
| Shape | Rhomb |

---
